# Furious lung abscess due to *Parvimonas micra*

**DOI:** 10.1002/rcr2.1161

**Published:** 2023-05-16

**Authors:** Shinnosuke Fukushima, Hideharu Hagiya, Hiromichi Naito, Fumio Otsuka

**Affiliations:** ^1^ Department of General Medicine Okayama University Graduate School of Medicine, Dentistry and Pharmaceutical Sciences Okayama Japan; ^2^ Department of Emergency, Critical Care, and Disaster Medicine Okayama University Graduate School of Medicine, Dentistry, and Pharmaceutical Sciences Okayama Japan

**Keywords:** bloodstream infection, lung abscess, *Parvimonas micra*, pneumonia

## Abstract

*Parvimonas micra* commonly present in the oral cavity and intestinal tract of humans. *P. micra* indicating a high virulence, has the potential of forming abscess. The infection of *P. micra* may require surgical excision.

## Graphical abstract

We present a case of lung abscess due to *Parvimonas micra* in a 57 year old woman who did not respond to antibiotics and needed surgical resection, indicating the high virulence of this bacteria.

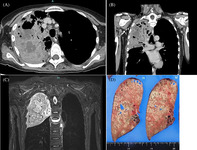

## CLINICAL IMAGE

**FIGURE 1:**
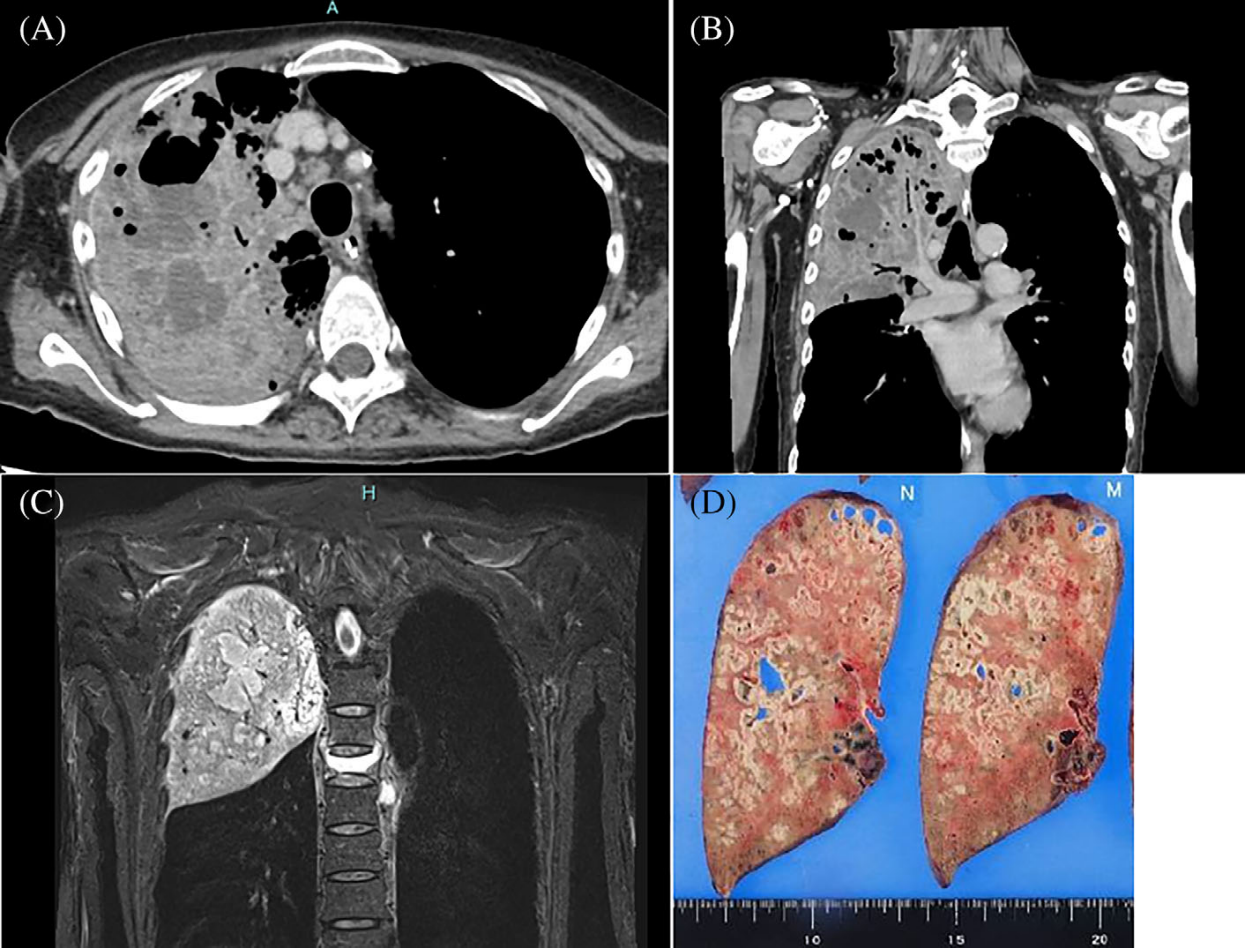
Multilocular lung abscess of the right upper lobe. Contrast‐enhanced computed tomography (A, axial; B, coronal) and magnetic resonance imaging (C; T2 weighted image) demonstrated the involvement of a multilocular lung abscess in her right upper lobe. The resected upper lobe showed the involvement of a multilocular lung abscess (D; macro picture).

A 57‐year‐old Japanese woman with bipolar disorder was transported to our emergency room in a shock state. She was intubated and hospitalized in an intensive care unit. Contrast‐enhanced computed tomography and magnetic resonance imaging revealed a multilocular lung abscess of the right upper lobe (Figure 1) and blood culture detected *Parvimonas micra*, and revealed the minimum inhibitory concentration of the antibiotics (Table 1). We treated the patient with ceftriaxone, vancomycin, and azithromycin, but high fever along with circulatory failure persisted. She underwent a right upper lobectomy on the 10th admission day to eliminate the infectious focus (Figure 1). The pathological investigation showed atelectatic tissues occupied with exudates, histiocytes, and monocytes. She had a favourable clinical course postoperatively.

**TABLE 1:** The minimum inhibitory concentration (MIC) of the antibiotics.

Antibiotics	MIC
Penicillin G	≦0.06
Sulbactam/Ampicillin	≦0.5
Cefazolin	≦0.5
Cefmetazole	≦0.5
Cefepime	≦0.5
Imipenem	≦0.5
Levofloxacin	4
Gentamicin	≦2
Arbekacin	>8
Minocycline	≦4
Erythromycin	>4
Clindamycin	>4
Vancomycin	≦0.5
Teicoplanin	≦0.5
Daptomycin	1
Linezolid	1
Sulfamethoxazole‐Trimethoprim	>40
Rifampicin	≦1
Oxacillin	≦0.25
Cefoxitin	≦4

*P. micra*, alternatively is a gram‐positive cocci, commonly present in the oral cavity. Purulent diseases are typical pathologies of *P. micra* infection, and patients with lung abscess due to *P. micra* were reported in elderly men with poor oral hygiene who smoke and drink heavily.^1^, ^2^ However, the present patient had no such risk factors. The patient did not respond to the antibiotics given and only improved after the surgical intervention indicating the high virulence of this bacteria. The patient was discharged with a prescription of amoxicillin clavulanic acid for 10 weeks of antibiotic therapy.

## AUTHOR CONTRIBUTIONS

Shinnosuke Fukushima and Hiromichi Naito contributed to patient care. Shinnosuke Fukushima wrote the manuscript, Hideharu Hagiya revised, and Fumio Otsuka organized it.

## CONFLICT OF INTEREST STATEMENT

None declared.

## ETHICS STATEMENT

Written informed consent was obtained from the patient to publish this case report.

## Data Availability

Data not shared.
